# Two case reports of right atrial aneurysm

**DOI:** 10.1097/MD.0000000000019748

**Published:** 2020-04-17

**Authors:** Hao-Peng Li, Xian-Wang Ye, Hai-Tao Wang

**Affiliations:** a Department of Radiology, Ningbo Women and Children's Hospital; b Department of Radiology, Ningbo No. 1 Hospital; c Department of Vascular Intervention, The Affiliated Hospital of Medical School of Ningbo University, Ningbo, Zhejiang, China.

**Keywords:** aneurysm, right atrial aneurysm

## Abstract

**Introduction:**

Right atrial aneurysm (RAA) is a rare congenital heart disease (CHD) that usually shows no symptom and is discovered occasionally. This paper introduces the clinical and imaging data obtained in 2 RAA patients and presents a related literature review with the aim of increasing understanding of this disease.

**Patient focus:**

One case showed chest distress, while the other showed symptoms on physical examination and positive signs.

**Diagnosis:**

Both of these 2 cases were diagnosed with RAA based on ultrasonography, computed tomography angiography (CTA), and enhanced magnetic resonance imaging (MRI) examinations.

**Interventions:**

One patient was orally administered warfarin anticoagulant therapy, while the other was given amiodarone to control arrhythmia as well as warfarin anticoagulant therapy.

**Results:**

The clinical symptoms of both cases were not aggravated.

**Conclusions:**

RAA is a rare cardiac anomaly that can induce severe complications, and it is mainly diagnosed based on imaging examinations. Conservative treatment and regular imaging monitoring are recommended for asymptomatic patients with no high-risk factors, while surgical treatment should be performed in symptomatic patients with high-risk factors.

## 1 Introduction

Right atrial aneurysm (RAA) is a rare congenital heart disease (CHD); affected patients are likely to suffer from delayed diagnosis and treatment due to its hidden clinical symptoms. This paper reports the clinical and imaging data obtained in 2 RAA cases and reviews the related literature to enhance understanding of this disease.

## 2 Case report

**Figure 1:**
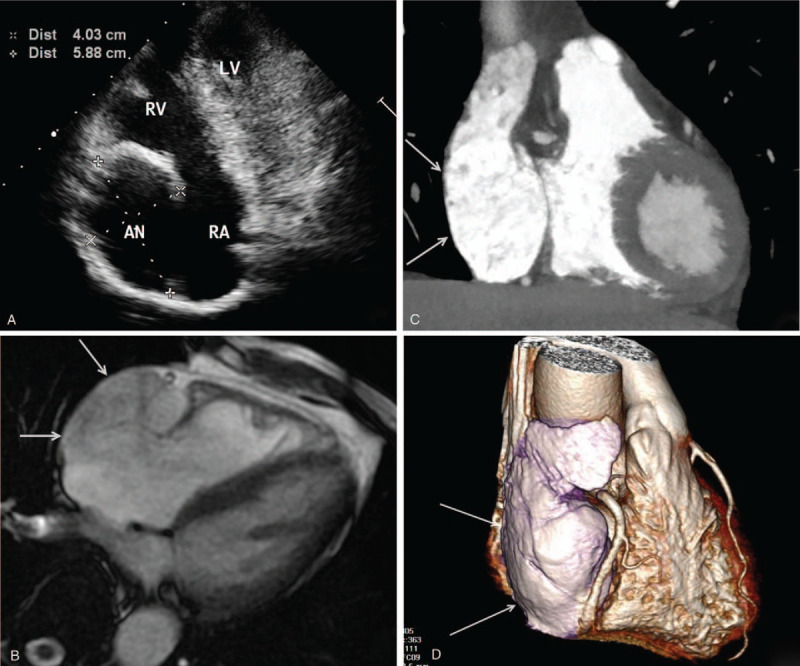
Ultrasonography, MR, and enhanced CT images obtained in case 1. (a) Ultrasonography revealed a cystic mass in the right edge of the heart, which was connected to the right atrium; (b) a diverticular lesion was seen inside the pericardium and found to be connected to the right atrium and to have a thin wall; (c and d) well-defined enhancement was observed in the localized extraneous lesion in the right atrium area, and a 3D image showed no external compressive structure in the adjacent coronary artery.

Patient 1 was a 61-year-old man who visited a local hospital 3 months prior due to chest distress. Chest fluoroscopy revealed a localized enlarged right lower edge, and he visited our hospital to seek a further diagnosis and treatment. An outpatient physical examination showed his body temperature was 36.5 °C, his pulse was 75 beats/minute, his blood pressure was 130/70 mm Hg, and his heart border was enlarged rightward. There was no other positive sign. An electrocardiogram revealed sinus rhythm with a ventricular rate of 80 beats/min and a regular heart rhythm. A 24-hour ambulatory electrocardiogram (AECG) revealed no supraventricular arrhythmia. Transthoracic echocardiography (IE33, PHILIPS, Netherlands) revealed a cystic mass 5.8 cm × 5.2 cm × 4.0 cm in size in the area of the right atrium (Fig. 1a); this mass was connected to the right atrium, and spontaneous angiography was observed within the mass. No thrombosis was seen in the mass, the tumor body compressed the right ventricle at diastole, and blood flow entering the right ventricle was not restricted. The left atrium and bilateral ventricles had normal structures, no abnormality was detected in the valve or ventricular wall activities, and the left ventricular ejection fraction was 73%. The patient was therefore diagnosed with RAA. Computed tomography (CT) angiography (CTA, Aquilion One, TOSHIBA, Japan) revealed a diverticular lesion in the right atrium area. Contrast agent filling was seen inside the lesion, and no external compressive structure was seen in the adjacent coronary artery (Fig. 1b and c). Magnetic resonance imaging (MRI, MAGNETOM Avanto 1.5T MRI, SIEMENS, Germany) also revealed diverticular changes in the right atrium area with a thin luminal wall (Fig. 1d). The patient had a 3-year history of hypertension, and his blood pressure was controlled at 120–140/70–90 mm Hg by orally administered antihypertensive drugs. The patient refused surgical treatment, so oral administration of warfarin anticoagulant therapy was prescribed, and the international normalized ratio of prothrombin time was maintained at 2.0 to 3.0.

**Figure 2:**
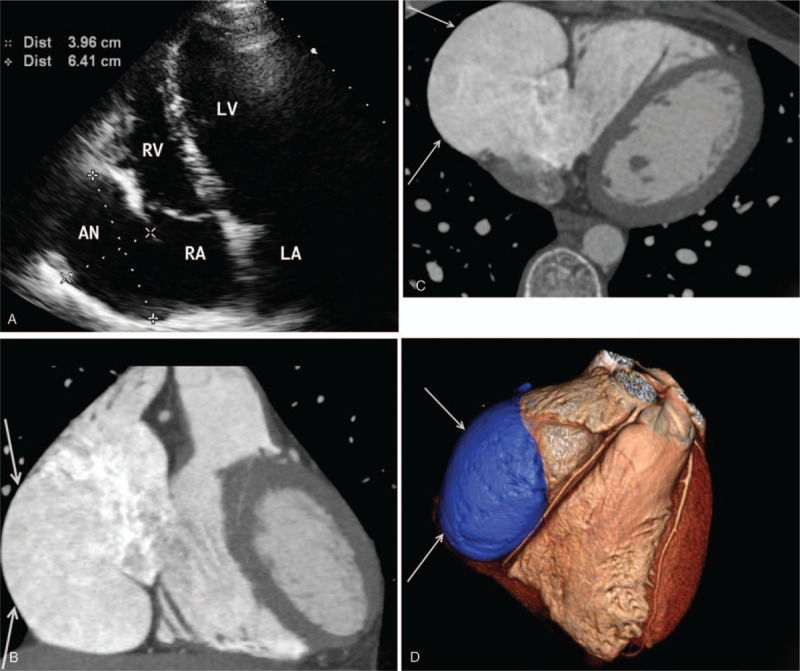
Ultrasonography and enhanced CT images obtained in case 2. (a) An ultrasound image revealed a cystic mass that connected the right edge of the heart with the right atrium. (b and c) Coronal and axial enhanced CT images revealed an oval-shaped diverticular lesion in the right atrium that had a thin wall and was connected to the right atrium. (d) A 3D image revealed no abnormality in the adjacent coronary artery.

Patient 2 was a 26-year-old man who visited our hospital due to the discovery of heart shadow enlargement during an orientation health check in another hospital 4 months prior. A physical examination revealed his body temperature was 36.7 °C, his pulse was 71 beats/minute, his blood pressure was 121/71 mm Hg, the heart border was enlarged bilaterally, and there was no detected cardiac murmur. ECG revealed type I atrial flutter with a ventricular rate of 77 beats/min, a regular heart rhythm, and that the cardiac electric axis was shifted leftward. Transthoracic ultrasonography (IE33, PHILIPS, Netherlands) revealed a cystic mass in the right auricle area (Fig. 2a) that was approximately 6.4 cm × 4.3 cm × 3.9 cm in size and was connected to the right atrium. No abnormality was observed in the bilateral ventricles or left atrial structure. Doppler ultrasonography revealed unrestricted bilateral ventricular blood filling at diastole. Transesophageal ultrasonography revealed dense spontaneous contrast agent stagnation inside the lesion, but no thrombosis was seen inside the lesion or in the left atrium. The patient was diagnosed with RAA. Chest CTA (Aquilion One, TOSHIBA, Japan) suggested that the right atrium was oval and protruded outside the cavity, and the contrast agent density inside the lesion was similar to that in the right atrium (Fig. 2b–d). The patient refused surgical treatment and was prescribed orally administered amiodarone and warfarin anticoagulant therapy. At present, the patient has been followed up for 4 months, and no clinical symptom has been observed.

## 3 Discussion

### 3.1 Epidemiology and pathogenesis

RAA is a rare congenital or acquired cardiac anomaly for which only just over 30 cases have been reported at home and abroad since the first report by Morrow et al in 1968.^[1]^ Currently, only approximately 20 cases have been reported worldwide. There are more male than female patients, and RAA can occur in all age groups, from fetal stages to adulthood.^[2]^ Congenital RAA is caused by dysplasia of the muscular wall in the right atrium,^[3]^ while the pathogenesis of acquired RAA remains unclear and is speculated to be caused by the long-term action of diseases such as pulmonary artery hypertension, CHD, and heart failure (HF), which increase right atrial pressure and enlarge the right atrial volume.^[4]^ A tiny minority of patients may be complicated by left atrial aneurysm.^[5]^ The histological manifestations of RAA include endocardial fibrosis and fatty degeneration with no endocardial or myocardial inflammatory reaction.^[6]^

### 3.2 Clinical features and complications

According to a literature report, 48% of RAA patients are showing no symptoms when the disease is discovered, although 28%, 17%, and 12% of patients develop polypnea, palpation, and arrhythmia, respectively, and a tiny minority of patients may have right HF and easy fatigability.^[7]^ In addition, some patients develop symptoms of organ embolism due to thrombus detachment within the right atrium.^[2]^ Typically, heart border enlargement and rapid tachyarrhythmia are the most common signs observed in RAA patients, but most patients lack other positive signs. In addition, the complications of this disease include thrombosis, atrial tachyarrhythmia, right atrial rupture, and sudden death.^[8]^

### 3.3 Diagnosis

RAA should be diagnosed based on an imaging examination. Typically, transthoracic ultrasonography is the preferred examination, but the sensitivity of transthoracic ultrasonography as reported in the literature is as low as 12%.^[8]^ In contrast, transesophageal ultrasonography may improve diagnostic sensitivity. CT and MRI allow for a clear display of the relationships between RAA and surrounding structures, such as the right atrium and descending aorta, and achieves relatively high sensitivity and specificity, which are important in examinations for diagnosing RAA.^[9]^ Moreover, in RAA patients with a definite imaging diagnosis, a 24-hour AECG should be conducted to examine the presence/absence of atrial tachyarrhythmia.^[10]^

For asymptomatic RAA patients, conservative treatment through oral administration of anticoagulants and/or antiarrhythmic drugs should be the preferred choice.^[11]^ Typically, the former can prevent thrombosis and reduce thrombotic disease, while the latter can be used to treat atrial tachyarrhythmia as well as potential secondary ventricular arrhythmia and lower the incidence of complications. Such patients have a relatively good prognosis, but they should still be followed up closely; cardiac ultrasonography, CT, or MRI should be carried out periodically, and some scholars recommend re-performing a heart MRI every 2 years.^[12]^ Notably, concurrent CHD, RAA, and right atrial size are independent risk factors that affect prognosis^[9]^ and may increase the risks of complications, such as thromboembolism, atrial arrhythmia, and aneurysm rupture. Consequently, surgical treatments, such as atrial aneurysm resection and Maze III, should be implemented for patients combined with CHD, large RAA, or increased aneurysm size during follow-up, even if they present no clinical symptoms.^[9]^ For patients showing clinical symptoms, surgery should be the preferred therapeutic scheme.

## 4 Conclusions

RAA is a rare cardiac anomaly that can cause severe complications and is mainly diagnosed based on an imaging examination. Conservative treatment and regular imaging monitoring are recommended for asymptomatic patients with no high-risk factors, while surgical treatment should be performed in symptomatic patients with high-risk factors.

## Author contributions

**Investigation:** Xian-Wang Ye.

**Writing – original draft:** Hao-Peng Li.

**Writing – review & editing:** Hai-Tao Wang.
